# Pneumonia

**Published:** 1904-03

**Authors:** James B. Baird

**Affiliations:** Atlanta, Ga.


### PNEUMONIA.*

BY JAMES B. BAIRD, M.D.

ATLANTA, GA.

Pneumonia or pneumonitis is described as an acute infectious or an acute specific disease having its essential anatomical characteristics located in the lungs. It has been called, undoubtedly most appropriately, lung fever, which suggests a general or systemic disease with pulmonary manifestations.

The term infectious " denotes any causative agent which, under certain conditions, is capable of unlimited increase or multiplication. With this definition, an infectious disease may or may not be communicable by means of a contagium." A specific disease is one which depends for its development and propagation upon a specific or special cause.

*Read before the Atlanta Society of Medicine, February 4, 1904.

In the great majority of cases acute pneumonia begins with a well-defined chill. The invasion, as a rule, is abrupt. The period of incubation is not determined, but is probably very short. A sharp pain in the affected side, referred to the region of the nipple, is generally an early symptom. It may be severe, and is ascribed to coexisting pleuritis. Cough, short, frequent and dry, soon appears. The cough is sometimes wanting. It is frequently accompanied, after a few hours, by expectoration, at first scant, white, viscid; it may soon become "rusty," due to the intimate admixture with the secretion of a small amount of blood, causing the peculiar pinkish tinge. The sputum is very adhesive, so much so that it will remain in an inverted receptacle. A more abundant amount of dark-colored blood gives rise to the so-called "prune-juice" expectoration. This is usually found in adynamic cases.

Fever with the usual concomitants, namely, heat of the skin, lassitude, thirst, anorexia, headache and quickened pulse—promptly occurs. In mild cases the temperature usually does not exceed 104° F. A pyrexia in excess of this degree, probably indicates severity. The pulse varies from 80 to 120. The respirations are increased to 30 or 40 or more. This acceleration is due in part to the pain of full inspiration and the necessity for voluntary shortening of the act, and to the diminished breathing space requiring an increased number of respiratory efforts. A herpetic eruption is a very common early symptom of pneumonia. The vesicles may appear upon the lips, the nose, the anus or the genitals. A brisk hæmoptysis may be among the first symptoms. The invasion of a second or a third lobe is rarely accompanied by a chill.

The involvement of an entire lung is not always marked by symptoms denoting gravity. Frequently there is a conspicuous and surprising want of correspondence between the general symptoms and the local lesion. Even though an entire lung may be implicated, the fever may be slight, the respirations but little accelerated, and cough and expectoration may not be present.

The state of the urine affords indications of some importance. The quantity is reduced. The urea is in excess. The urates are deposited when it cools. Until resolution begins, the chlorides are generally reduced or are entirely absent. This phenomenon is not peculiar to pneumonia, the absence of the chlorides is observed in other diseases, but their return in these cases is an index of the commencement and the progress of resolution. A small amount of albumen is common, a large quantity indicates renal disease, which is confirmed by the presence of casts. Delirium, either mild or active, is not an unusual symptom. It is important in proportion to its persistence and prominence. A dusky, sharply defined redness of one or both cheeks is frequently observed. If this flush is limited to one cheek it does not necessarily correspond to the affected side. Icterus is sometimes present as a complication. It is attributable, in some cases, to slight duodenitis, and partial or complete closure of the common duct by tumefaction; but, occasionally, the jaundice is intense and its cause is obscure.

The term typhoid-pneumonia is used to indicate a low or depressed physical condition. It should not be confounded with pneumonia occurring in the course of typhoid fever. The fever frequently ends abruptly by what is called crisis. This abrupt decline in the temperature is the most remarkable phenomenon in connection with pneumonia. The defervescence is usually accomplished in several hours, but a fall of six or eight degrees has been known to occur in the space of one hour. The temperature may remain below normal during convalescence. If the fever should continue beyond twelve or fourteen days, the attack is apt to terminate gradually—by lysis, as this mode of decline is called. This course of the disease is more common in children than in adults.

The duration of an attack is from five to twenty-five days or more. The average duration is about twelve days. No period of life is immune from pneumonia. It may occur under five years and over sixty. The favorite period is between twenty and forty years. The ages of special susceptibility are early childhood, between twenty and forty years and over sixty. It occurs more frequently in winter and spring—especially in the latter—than in summer or autumn. No section of the country is free from its ravages. It has been claimed that it is more common and more severe in warm than in cold climates. So far as this oft-repeated assertion applies to our Southern States as compared with the

Northern States, its correctness may fairly be doubted. Acute pneumonia is apparently developed spontaneously. Exposure to cold may act as a contributing cause. Traumatism and debilitating influences may act in the same way. One attack is not protection against subsequent attacks. Indeed the reverse appears to be true. Recurrence is far more common than in any other acute disease.

There are numerous isolated instances on record favorable to the theory that pneumonia is communicable directly from one person to another, and some writers of prominence advise the disinfection of the sputa, evidently having the question of direct communicability—of a contagious virus—in mind, but the great preponderance of accumulated clinical evidence does not sustain the view that it is a contagious disease. Physicians, nurses, other members of a family and other inmates of a hospital ward in close attendance upon or in close proximity to the patient rarely contract the disease, and it seldom spreads through neighborhoods or entire communities, and when it does, the fact is due to its infectious and not to its contagious character.

The dependence of pneumonia upon a specific cause seems to be well established and generally accepted,and it is probably entirely correct to regard it as a specific, infectious disease and as a general systemic affection with special chest manifestations; in other words, as a lung fever, analogous to rheumatic fever, rather than as a local inflammation with associated symptomatic phenomena.

It is claimed by some pathologists that the pneumococcus of Frankel, described in 1884, is the sole cause of pneumonia ; others more guarded, assert that this micro-organism is *probably* the cause ot acute lobar as of broncho-pneumonia. It is found also in pleurisy, pericarditis, meningitis, peritonitis, synovitis, otitis, endocarditis and also in the dust and sweepings of rooms.

Infection most likely takes place through the bronchi, but it is probable that it may also occur through the medium of the blood. While various organisms are associated with the pneumococcus, notably the streptococcus, the staphylococcus and the pneumo-bacillus of Friedlander, discovered in 1883, it has not been conclusively shown that they are otherwise than secondary invaders.

Coplin says that bacteriological invasion, as a cause of acute lobar pneumonia is universally admitted. That it is, however, the result of a single organism seems to be no longer probable, although not absolutely disproved. Croupous pneumonia, he continues, which can not be differentiated either clinically or anatomically from the croupous pneumonia produced by the pneumococcus of Frankel, may result from infection by streptococcus and, possibly, by staphylococcus. The typhoid bacillus, the bacillus of influenza, colon bacillus, plague baccillus and probably a number of other micro-organisms may bring about that form of solidification usually regarded as anatomically characteristic of croupous-pneumonia.

The principal forms of acute-pneumonia are the lobar and the lobular. The former is sometimes called croupous or fibrinous, and the latter is styled catarrhal or broncho-pneumonia. Lobar pneumonia occurs chiefly in adults. The lobular variety is most frequent in young children, and is not uncommon in old persons.

For the purposes of this paper, it should be understood when the unqualified term pneumonia is used, it refers only to *lobar* pneumonia.

The disease manifests decided predilection for certain lobes. The lower lobes are its most frequent seat, and the right oftener than the left. The right upper lobe is more frequently invaded than the left. The morbid process may be confined to one lobe or, rarely, to a portion of one lobe, or it may involve a whole lung, the lower and upper portions being successively or, occasionally, simultaneously attacked. When corresponding lobes on opposite sides are attacked it is called double pneumonia, and crossed pneumonia when a lower and an opposite upper lobe are affected. It is desirable to understand that when a second or third lobe is attacked, it is not due to an extension of the inflammation but to the development of new points of infection.

In some cases the initial symptoms are distinctive, in others they are entirely latent. The chill, the pleuritic stitch, intense fever and rusty expectoration may all be absent. The physical signs then must be relied upon to establish the diagnosis. These signs in the first stage—during the period of engorgement—are moderate dullness and the crepitant rale. This rale is not always present and delay may then be necessary. The crepitant rale and the rusty sputa are regarded as pathognomonic. This sign is available only in the first and latter stages of an attack. The sound is characterized by its fineness, dryness and limitation to inspiration. Care should be taken not to confound the crepitant rale with the subcrepitant rale. The subcrepitant rale is a fine, but moist and bubbling sound heard with both acts of respiration and is bilateral. The peculiar sound known as the crepitant rale is probably produced by the separation of the sides of the hyperæmic air-cells, though it has been seriously suggested that it is caused by a fine pleural friction. This opinion, in my judgment, is untenable. The rale is comparatively infrequent in lobes secondarily attacked.

As solidification progresses, this sign is lost, and the cells being partially obstructed or entirely impervious to air, only the sounds from the bronchial tubes are heard. These sounds are not reverberant, and the broncho-vesicular murmur or the bronchial or tubular breathing results. The consolidated tissue transmitting readily to the ear the voice sounds, broncophony, both of the spoken and whispered voice, is heard, and the dullness or flatness on percussion over the solidified area is found.

Pleuritis, with fibrinous exudation, is always present over the diseased lobe, when the inflammation of the lung tissue affects the surface of the lobe, which it does except in very rare instances. Copious effusion into the pleural cavity seldom occurs. When it does take place, in appreciable amount, the disease is designated pleuro-pneumonia. Circumscribed bronchitis—that is, bronchitis in the affected lobe—is usually present. General bronchitis may coexist. But neither disease manifests any tendency to eventuate in the other.

For convenience, the course of pneumonia is divided into stages. First, the stage of congestion or engorgement, marked by a hyperæmic state of the pulmonary capillaries and the presence of a bloody serum in the air-cells. This stage usually continues from twenty-four to forty-eight hours—exceptionally shorter than twenty-four or longer than forty-eight.

The second stage is characterized by solidification, called, also, hepatization, from the deposit within the cells of an inflammatory exudation. The duration of this stage may be fixed at two to four days.

The third stage embraces the period of resolution. This may run from three or four to eight or ten days or longer; occasionally, several weeks elapse before the deposit is entirely absorbed. If the disease should pass into the purulent stage, death may occur in a few days, or recovery may slowly ensue, and many weeks may elapse before the normal condition is restored.

The most frequent complications of pneumonia are—pleuritis, pericarditis, endocarditis, meningitis, icterus and albuminuria; peritonitis is rare. The cerebral symptoms in children are sometimes so pronounced that attention is diverted from the chest, and suspicion rests solely upon the brain as the seat of the offending lesion.

The general symptoms, supplemented and fortified by the physical signs, leave, as a rule, little room for doubt as to diagnosis.

As a summary of the essential clinical characteristics, the following paragraph by the elder Dr. Flint is complete:

"It is a fever characterized anatomically by an abundant exudative deposit in the air-vesicles of a single lobe, or of two, and sometimes three lobes of the lungs, with, in general, circumscribed bronchitis and dry pleurisy. It is a fever which rapidly reaches its maximum intensity, and has a short career—the duration averaging about eleven days. It proves fatal chiefly in consequence of associated diseases, complications or accidents, and in the mode of dying, asthenia usually predominates. It depends on a cause or on causes, specific in character, the nature of which is not at present established. It sometimes aborts spontaneously, and it is, in some instances, arrested by remedies. If not arrested, it may be favorably modified, its duration abridged and the danger to life diminished by treatment addressed, not to the pulmonary affection, but to the fever."

The prognosis is very variable—widely collected statistics show an average fatality of about eighteen per centum. Pneumonia stands first as a cause of death from all acute diseases. In some sections of our country it has, within the last decade, usurped the place so long held by consumption, and it now leads the list of mortality. The prognosis is unfavorable for recovery in old and feeble persons, in alcoholics, in cases of weak hearts and defective kidneys. In young subjects of fair vigor, uncomplicated cases, not of excessive intensity or of unusual extent, tend toward recovery.

Toxæmia is a most important prognostic feature. It outranks pyrexia and consolidation as a dangerous factor. Death from failure of the respiratory function is rare. Weakness of the heart is a momentous element. This tendency is due to the direct debilitating effect of the peculiar poison acting by its depressing influence through the nervous system, to sustained high temperature, or to over-distention of the right cavities—either or all of these causes impair, more or less, the integrity of the cardiac muscle. Asthenia may be rapid—simulating closely collapse—though the decline in strength is usually gradual.

In the treatment of pneumonia we have no specific. The general indications call for support. In his reference to abortive treatment of the disease, Dr. Flint, in the paragraph quoted above, had reference to the early administration of quinine in doses of twenty to forty grains—given at once or distributed through several hours. It has not seemed to me that subsequent experience with the drug sustains this view.

Purgatives, more or less active, as eliminants are indicated through the course of the disease. Blood-letting has its appropriate place among useful therapeutic measures, and when judiciously employed, it is capable of prompt and decided benefit, though it is seldom necessary, never, except in the very first stage, and then, only in a few suitable cases.

Veratrum viride, formerly extensively employed, is now comparatively rarely used. It has been displaced by newer drugs. It is undoubtedly a valuable resource, and deserves more credit than it now receives. Tartar emetic, given for its sedative and expectorant effects, has likewise fallen into disuse. Opium holds an important place in the therapeutics of this as of many other diseases. Its power to allay pain, soothe agitation and quiet cough renders it indispensable in the great majority of cases. It should be used with circumspection when bronchorrhœa or extensive hepatization exists. Expectorants, as muriate or carbonate of ammonia, are not indicated. The exudation is not removed by expectoration, but by absorption, and the unnecessary multiplication of remedies should, for the patient's sake, be avoided. Drug antipyretics, in my experience, are useful. I prefer phenacetine to other preparations of this class. Given in moderate doses—two or three grains, I have never seen any bad results or any appreciable depression from its use. On the other hand, it should be classed among the supporting measures, as it contributes directly to the conservation of strength by promoting comfort and by saving the nervous system and the heart from the exhausting effects of prolonged high temperature.

Hydrotherapy is a useful adjuvant—sometimes an effectual substitute. I generally employ the tepid partial sponge bath; sometimes the hot bath is efficient. Ice-bags to the head are useful and ice coils or bags to the chest are recommended. Recognizing the tendency toward asthenia, stimulants should be given early and in many cases freely. The necessity for support should be anticipated and asthenia, if possible, should be forestalled. Very large doses of whisky are sometimes well borne. The doses should be gradually increased and apportioned to the effect upon the pulse both as to its frequency and quality, and upon the general system. The carbonate or the aromatic spirit of ammonia are serviceable for their stimulating properties. Moderate doses of strychnine should be used throughout the attack. Digitalis may be added if the pulse begins to flag. Hypodermic injections of camphor in ether should be administered to avert threatened collapse. Sinapisms, iodine or dry cups will check pleuritic pain. Blisters, in my judgment, are only admissible in the last stage to encourage delayed or very tardy resolution. Oxygen gas is of doubtful utility, yet, under certain conditions, it may be cautiously used.

The indications for saline infusions are not peculiar to pneumonia. A suitable diet and sometimes hypnotics are called for. It is barely possible that the adrenaline chloride may prove of some value by lessening congestion of the bronchial tubes in the unaffected lobes.

